# Supplementary material for: A Vibronic Coupling Model to Study the Nonadiabatic Dynamics of Polyenes
Source: J Phys Chem A. 2026 Mar 18;130(13):2765–77. doi: 10.1021/acs.jpca.6c00402 (PMC13051437; doi:10.1021/acs.jpca.6c00402)
Supplement: Supplementary file 1 [file jp6c00402_si_001.pdf]

A Vibronic Coupling Model to Study the Nonadiabatic Dynamics of Polyenes: Supporting Information

Timothy N. Georges,[*,†,‡] Louis Summerley,[†,¶] Johan E. Runeson,[§] and William Barford[*,†,‡]

†*Department of Chemistry, Physical and Theoretical Chemistry Laboratory, University of Oxford, Oxford OX1 3QZ, UK*

‡*Balliol College, University of Oxford, Oxford OX1 3BJ, UK*

¶*Worcester College, University of Oxford, Oxford OX1 2HB, UK*

§*Institute of Physics, University of Freiburg, Freiburg 79104, Germany*

E-mail: timothy.georges@chem.ox.ac.uk; william.barford@chem.ox.ac.uk

1 Parameterization

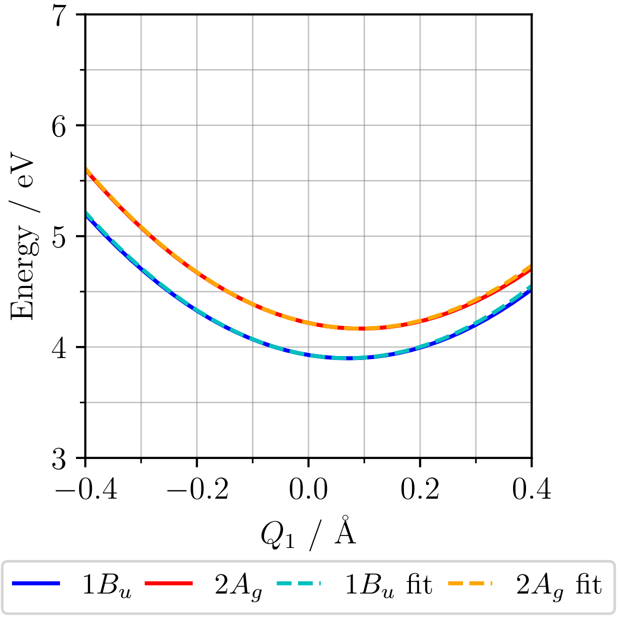

Figure S1: Cuts of the excited-state potential energy surfaces along the lowest-energy symmetric mode, Q_1.

Figs. S1 to S4 show the calculated potential energy surfaces on each mode and the fitted PES of the two-state LVC Hamiltonian. The highest-energy symmetric mode, Q_5, is shown in the main text.

Next we give the Hamiltonian parameters for dimensionless coordinates, where $\tilde{\kappa}_\alpha^{(i)} = \kappa_\alpha^{(i)} l_\alpha$ and $\tilde{\lambda}_\alpha^{(i,j)} = \lambda_\alpha^{(i,j)} l_\alpha$. The length scale is $l_\alpha = (\hbar/m\omega_\alpha)^{\frac{1}{2}}$. The two-state hexatriene LVC Hamiltonian has energies $E^{(1)} = 3.92795$ eV and $E^{(2)} = 4.21704$ eV. The rest of the parameters are given in Table S1

Table S1: Parameters of the linear vibronic coupling Hamiltonian of hexatriene. All values are in eV. Copied from Table 1 in the main text

Mode, α	1	2	3	4	5
ω_α	0.07203	0.13801	0.19692	0.22524	0.24041
$\tilde{\kappa}_\alpha^{(1)}$	−0.06433	—	0.01340	—	−0.34202
$\tilde{\kappa}_\alpha^{(2)}$	−0.08505	—	0.17861	—	−0.64464
$\tilde{\lambda}_\alpha$	—	0.03267	—	0.08692	—

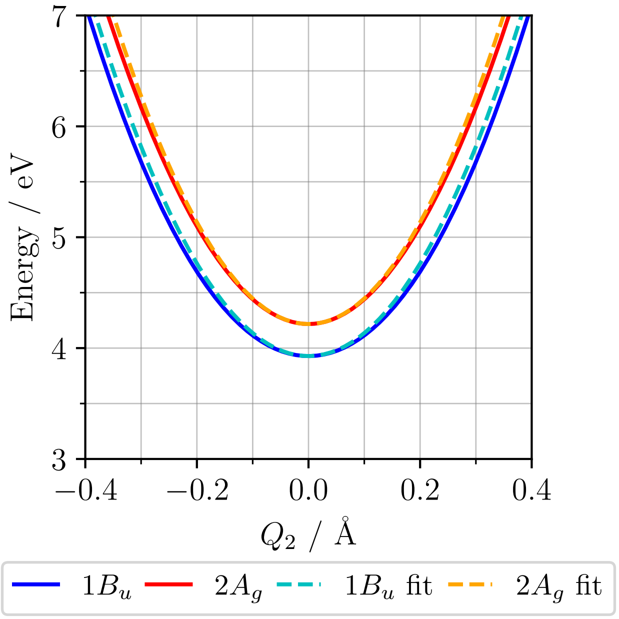

Figure S2: Cuts of the excited-state potential energy surfaces along the lowest-energy antisymmetric mode, Q_2.

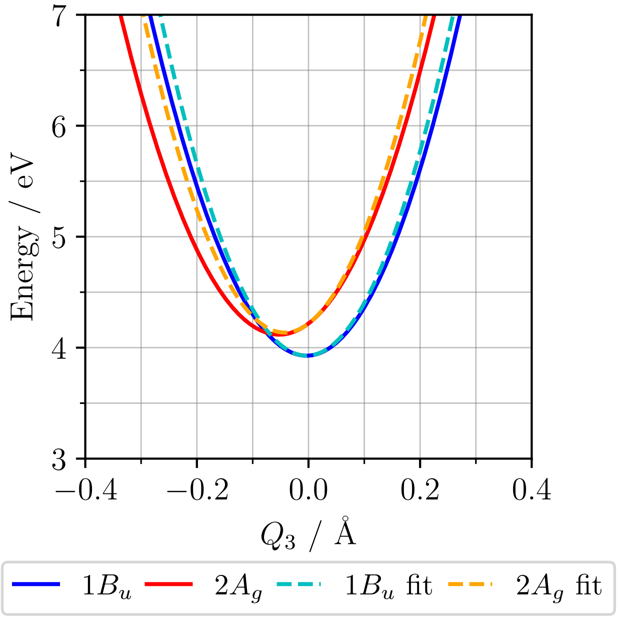

Figure S3: Cuts of the excited-state potential energy surfaces along the symmetric mode, Q_3.

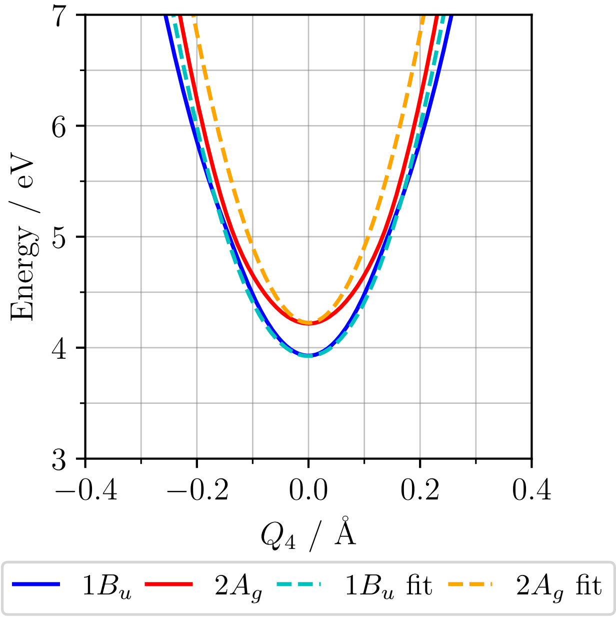

Figure S4: Cuts of the excited-state potential energy surfaces along the highest-energy anti-symmetric mode, Q_4.

All parameters for the extended Hubbard-Peierls Hamiltonian are given in the main text, except the particle-hole symmetry-breaking term, ϵ_n and ground-state equilibrium geometry. We find the following values (given in eV)

$$\epsilon = (1.702, 0.473, -1.982, -1.982, 0.473, 1.702), \tag{S1}$$

converge the carbon charge densities of our extended Hubbard-Peierls DMRG calculations to Mulliken charge densities found with DFT. The ground-state equilibrium bond-hopping integrals in eV are

$$t = (2.604, 2.118, 2.557, 2.118, 2.604). \tag{S2}$$

2 Convergence

2.1 Short Iterative Lanczos Propagator Method

For a given Hamiltonian, results with SILP should agree exactly with the multi-configurational time-dependent Hartree (MCTDH) method which is described below. This comparison is shown in Fig. S5, for the population dynamics of the two-state hexatriene LVC Hamiltonian. The SILP simulation was run with 30 Lanczos vectors and an adaptive time step threshold of $\epsilon = 10^{-8}$. The number of quantum harmonic oscillator levels in each mode, D_α, was $(14, 9, 12, 12, 19)$.

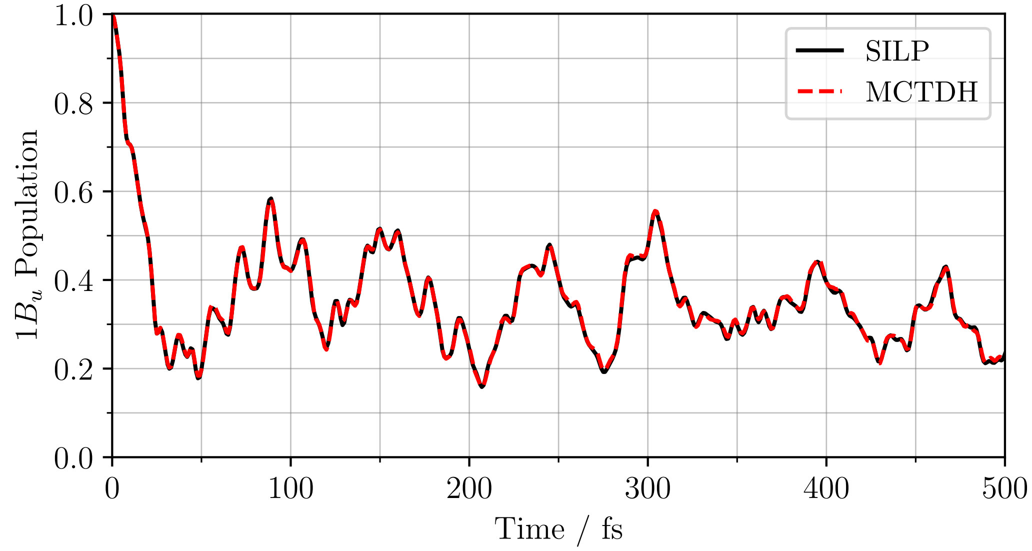

Figure S5: A comparison of the $1B_u$ population against time for the two-state hexatriene LVC Hamiltonian, for MCTDH and SILP. The overlapping curves shows the two methods are converged.

In the MCTDH method, a multi-configurational Hartree product of a small number of single particle functions are evolved through time according to the Dirac-Frenkel time-dependent variational principle. The time-dependent single particle functions are expanded in turn in a time-independent discrete variable representation of harmonic oscillator eigenfunctions. For details of the algorithm and calculation of observables we refer the reader to the following references.[1–4] We performed MCTDH calculations using the QUANTICS

package.[5,6] We use a starting state of $1B_u$ in the ground state geometry.

The calculations were converged using 8 single particle functions per electronic state in the multi-set formalism, with a primitive basis of quantum harmonic eigenfunctions in the discrete variable representation. For the two-state hexatriene LVC Hamiltonian, we found the following number of quantum harmonic oscillator functions per mode kept end of grid maximum populations below 0.000015: $(25, 15, 20, 20, 35)$.

2.2 Quantum-Classical Methods

All quantum classical methods must be converged in the time step, Δt, and the number of trajectories, N_T. To show this, we compare three different parameter sets against a reference set. The four parameter sets are:

- **a**: $\Delta t = 0.05$ fs, $N_T = 10^6$
- **b**: $\Delta t = 0.05$ fs, $N_T = 10^5$
- **c**: $\Delta t = 0.5$ fs, $N_T = 10^6$
- Reference: $\Delta t = 0.005$ fs, $N_T = 10^5$

For the two-state hexatriene LVC Hamiltonian, the difference in the $1B_u$ population in time against the reference parameter set is shown in Figs. S6 to S8. MTE requires a time step of 0.05 fs to converge to within 1% of the reference parameter set at all times. MASH converges the most readily and a time step of 0.5 fs lies within 3% of the reference parameter set. INT-FSSH struggles to converge at short times when the curve crossing is passed through, but long-time populations converge well.

To confirm this, we calculate the root mean square deviation of each simulation from the reference simulation. These results are shown in Table S2. This shows numerically that for a time step of 0.05 fs, INT-FSSH is the most poorly converged, while for a longer time step of 0.5 fs MTE converges the most poorly. For MASH, the 0.5 fs simulation converged better

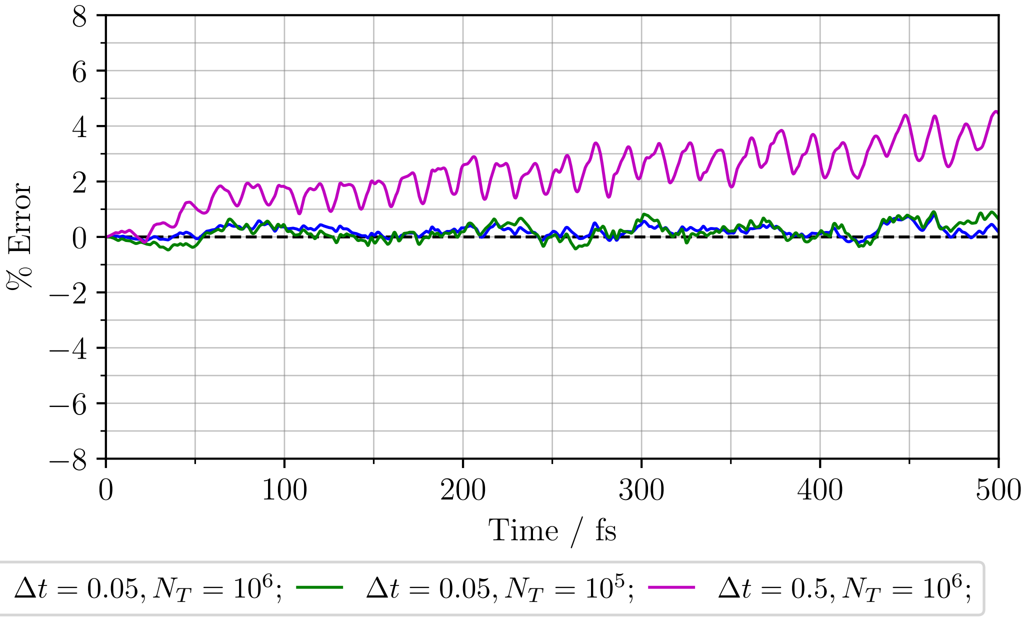

Figure S6: Population convergence test for MTE. A time step of 0.05 fs is converged within 1% of the reference parameter set. A longer time step of 0.5 fs is not converged.

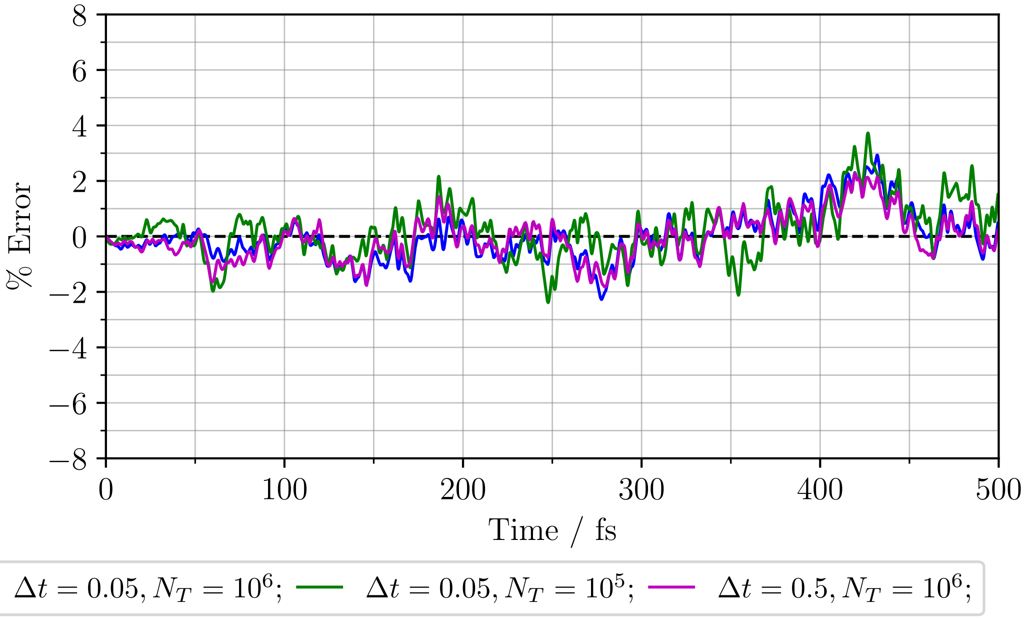

Figure S7: Population convergence test for MASH. MASH readily converges to the reference parameter set even with a time step of 0.5 fs, to within 3%.

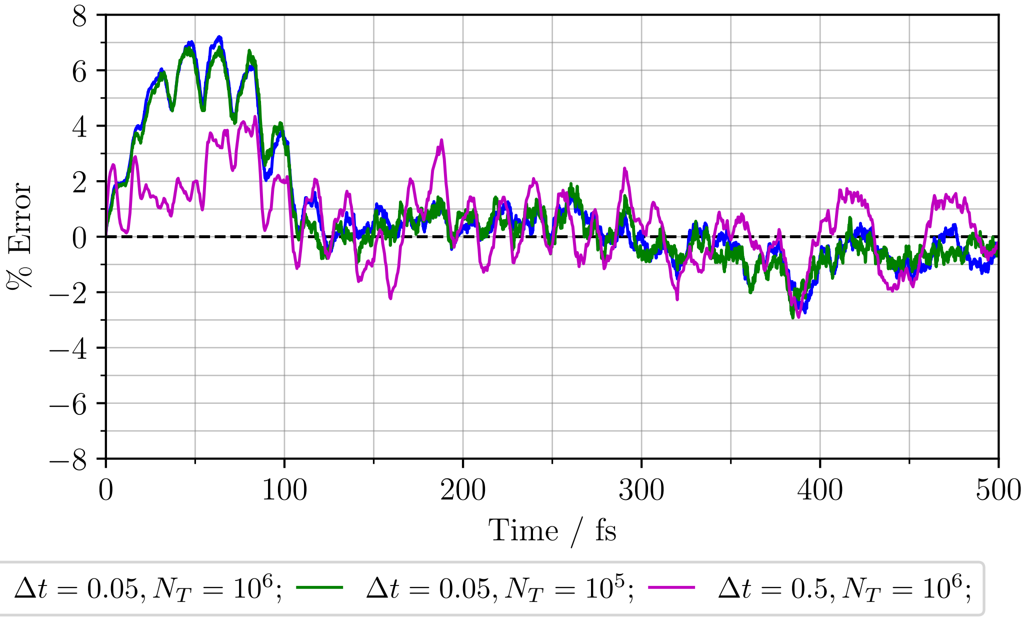

Figure S8: Population convergence test for INT-FSSH with $r_0 = 0.01$. A time step of 0.05 fs poorly converges to the reference parameter set below 100 fs. Therefore, we use the reference parameter set for INT-FSSH, which has a time step of 0.005 fs.

than the simulation with 10^5 trajectories and a smaller time step of 0.05 fs, highlighting the importance of having lots of trajectories when sampling both nuclear and electronic phase spaces.

Table S2: Root mean squared deviation of the $1B_u$ population against time, for the two-state hexatriene LVC Hamiltonian. The reference simulation has a time step of $\Delta t = 0.005$ fs and $N_T = 10^5$ trajectories. Column **a** are the parameters used in the paper for MTE and MASH. For INT-FSSH we used the reference parameter set, because of the large deviations in $1B_u$ population before 100 fs seen with the longer time step of 0.05 fs.

	**a**	**b**	**c**
**Method**	$\Delta t = 0.05$ fs, $N_T = 10^6$	$\Delta t = 0.05$ fs, $N_T = 10^5$	$\Delta t = 0.5$ fs, $N_T = 10^6$
MTE	0.001358	0.001421	0.010426
MASH	0.001256	0.002238	0.001512
INT-FSSH	0.008062	0.008258	0.003942

Following these investigations, we decided parameter set **a** was suitable for MTE and MASH simulations in the main paper. For INT-FSSH, we use the reference parameter set, because a larger time step of 0.05 fs leads to large deviations in $1B_u$ population before 100 fs.

3 FSSH Observables

There are multiple ways to define adiabatic populations in FSSH. One way is to use the wavefunction coefficients like in MTE, so $P_a = |c_a|^2$, which we refer to as 'wavefunction' observables. The other method is to set P_a equal to the proportion of trajectories on active state a, which we refer to as 'active state' observables. Fig. S9 shows the diabatic populations found with both population measures. The two measures differ when no decoherence correction is included, but are consistent when the INT correction is used.

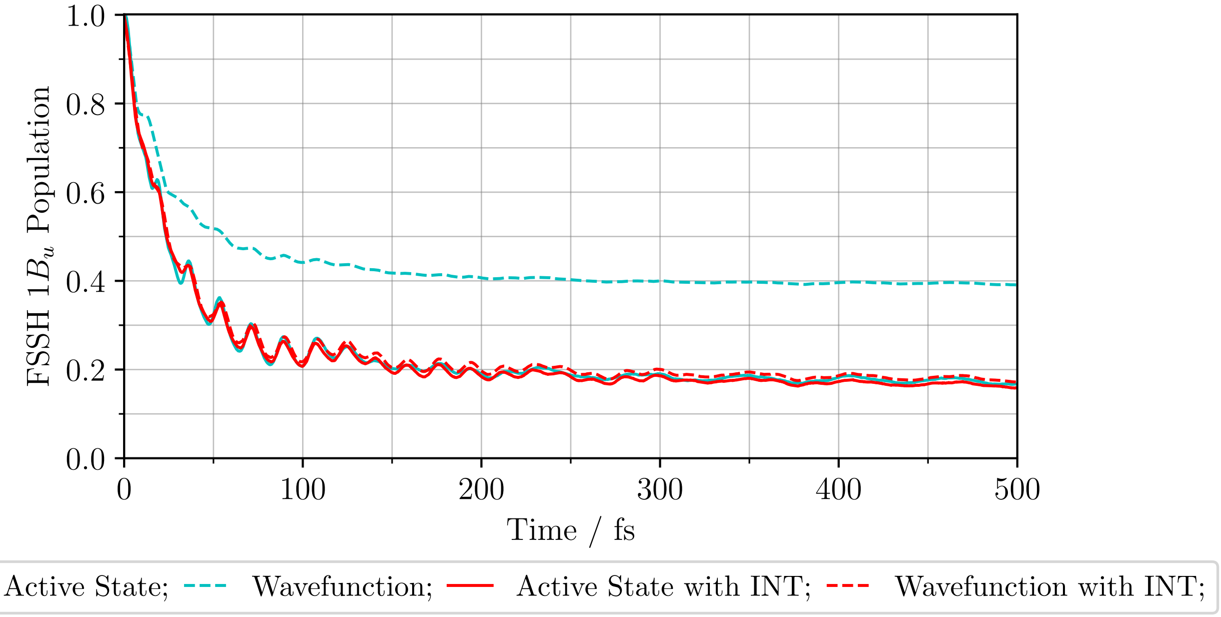

Figure S9: FSSH diabatic populations against time found via two different methods, with and without the INT decoherence correction. The decoherence correction makes the two approaches consistent.

4 The Role of the Q_1 Mode

It was shown in Fig. 3 of the main paper that the quantum-phonon SILP result has long-time oscillations in the population, with a period of 60 to 80 fs. This is a similar period to the 58 fs oscillations in Q_1, shown in Fig. 5 (a) of the main paper. To test whether the Q_1 mode causes these population oscillations, we performed a SILP simulation where we removed the

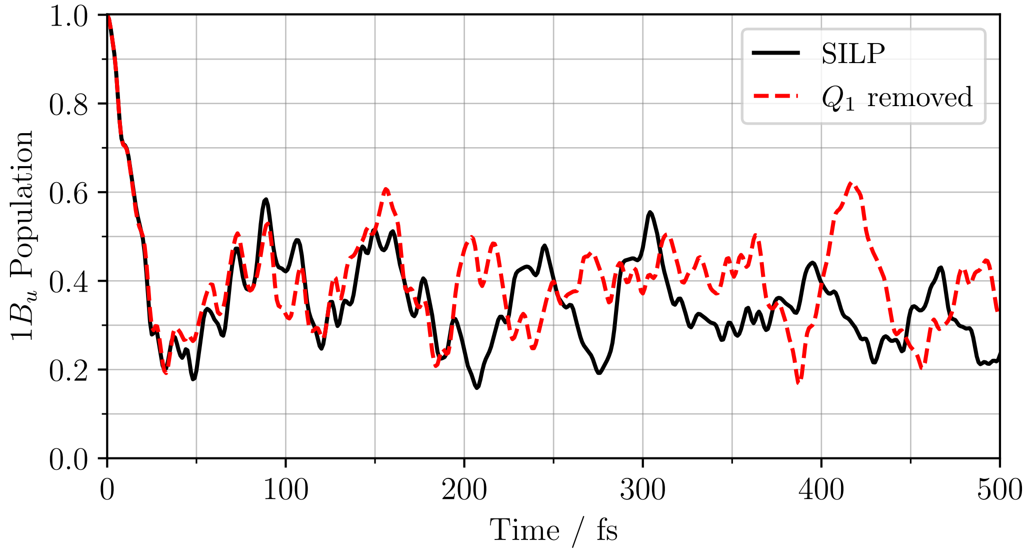

Figure S10: SILP diabatic populations against time, plotted for the full LVC Hamiltonian, and with the Q_1 mode removed. The 60 to 80 fs period oscillations remain.

Q_1 mode. As shown in Fig. S10, the 60 to 80 fs oscillations remain when Q_1 is removed. In fact, the Q_1 mode has little effect at all on the population dynamics up to 200 fs. Therefore, these oscillations are due to complicated vibronic interference that the quantum-classical methods used in this paper fail to describe.

5 Parameter Scan

For the parameter scan, we used a SILP adaptive time step error of $\epsilon = 10^{-8}$ and a Lanczos space of $N_L = 25$. For the ΔE scan, we used $D_\alpha = (14, 9, 12, 12, 19)$ levels. For the $\kappa_5^{(2)}$ and K_5 scans, we used $D_\alpha = (12, 7, 10, 10, 25)$ levels. For the λ_2 scan, we used $D_\alpha = (12, 9, 10, 12, 17)$ levels. Fig. S11 shows the standard deviation in the long-time $1B_u$ population P_∞ across four parameters scans. SILP has greater standard deviation throughout, because of its quantum oscillations. All quantum-classical errors lie below 0.0006 and all SILP errors lie below 0.0075. That is, except for the data point at $\kappa_5^{(2)} = -8.8$ eVÅ^{-1}. The exponential fit is very poor for this data point. Therefore, we remove this anomaly from Fig. 6 (b) of the main paper.

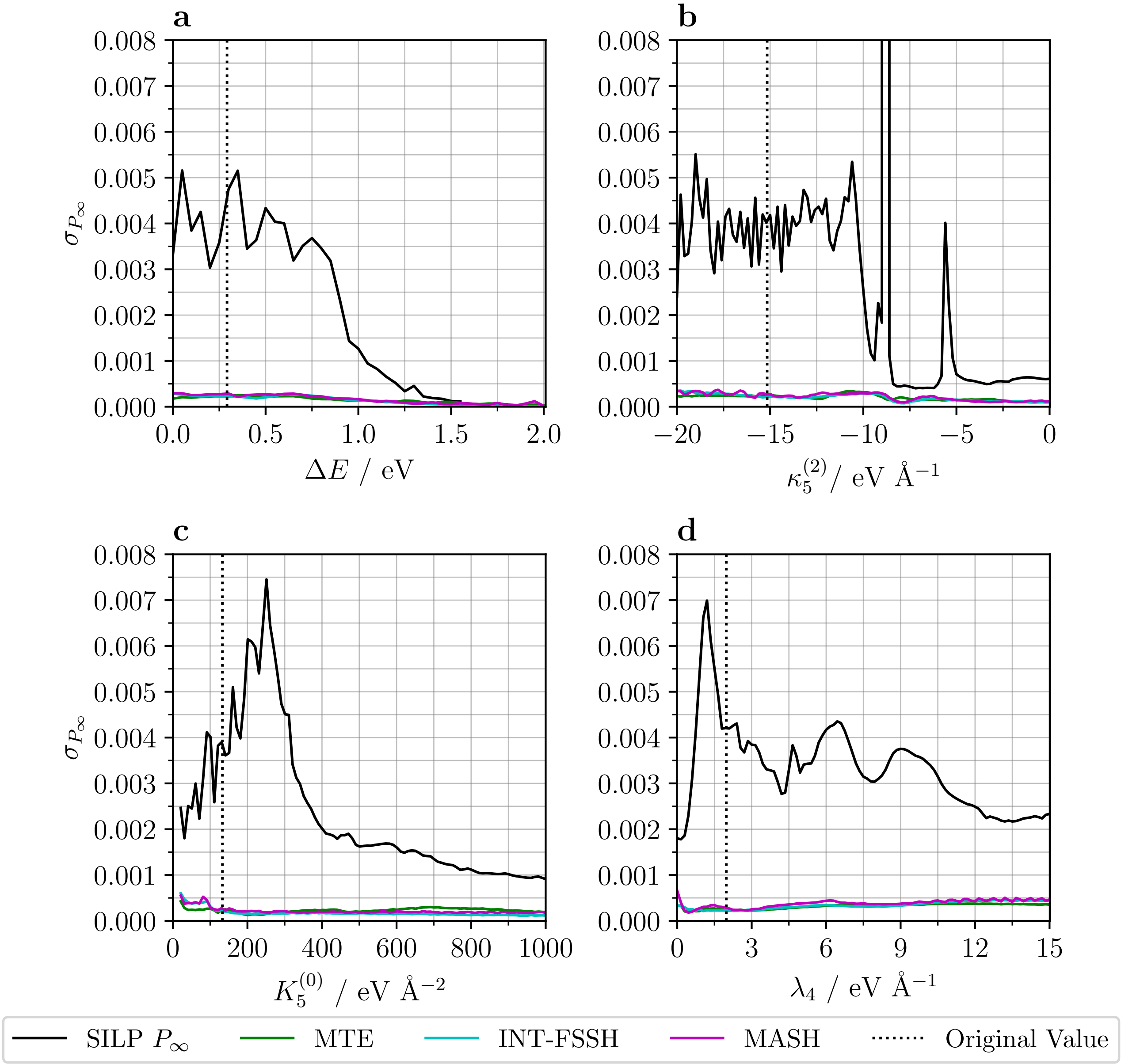

Figure S11: Plots of the error in the long-time $1B_u$ population, P_∞ for a range of parameters. This parameter converges well for all parameter regimes - SILP always lies below 0.0075 and the quantum-classical methods are always below 0.0006. The anomalous point in panel (b), caused by a poor exponential fit, is removed from Fig. 6 (b) of the main paper.

To confirm that P_∞ is a reliable measure of long-time average populations, we compare it to taking the mean of the $1B_u$ population in different time ranges. The time ranges chosen were from 200 to 350, 400, 450 and 500 fs. As shown in Fig. S12, these methods agree closely in almost all parameter regimes. This confirms that the oscillations with respect to parameter in each of the plots is physical and not caused by poor fitting. The two measures of long-time population disagree most in Fig. S12 (b) below -17.5 eV Å. However, both approaches support the conclusion that MTE is more accurate than the surface-hopping methods in this area of the parameter scan.

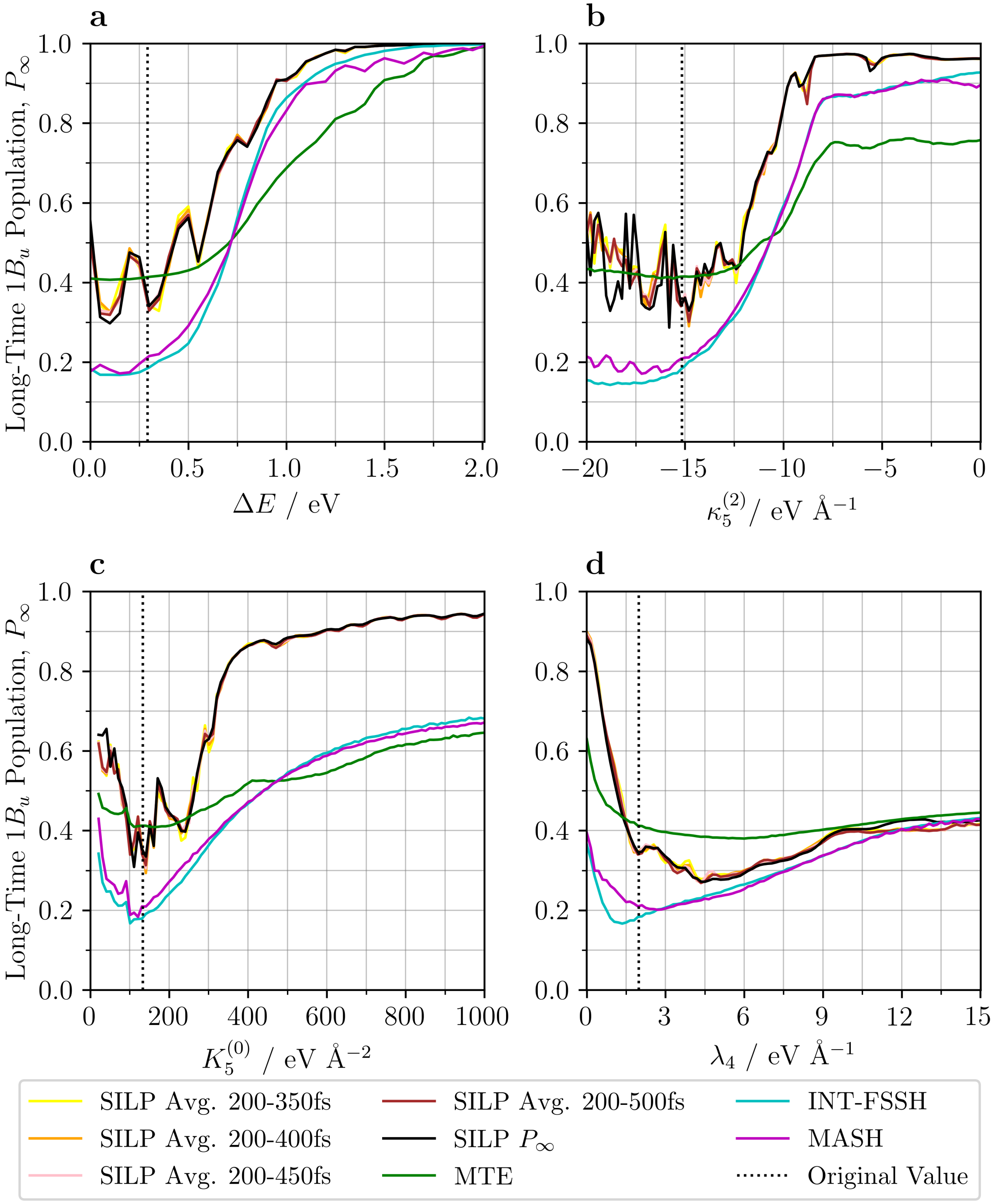

Figure S12: Plot of long-time populations using different measures for SILP. Taking the mean of populations in different time ranges gives similar results to finding P_∞ from an exponential fit.

References

(1) Meyer, H.-D.; Manthe, U.; Cederbaum, L. S. The Multi-Configurational Time-Dependent Hartree Approach. *Chemical Physics Letters* **1990**, *165*, 73–78.

(2) Manthe, U.; Meyer, H.-D.; Cederbaum, L. S. Wave-Packet Dynamics within the Multiconfiguration Hartree Framework: General Aspects and Application to NOCl. *The Journal of Chemical Physics* **1992**, *97*, 3199–3213.

(3) Worth, G. A.; Meyer, H.-D.; Cederbaum, L. S. The Effect of a Model Environment on the $S2$ Absorption Spectrum of Pyrazine: A Wave Packet Study Treating All 24 Vibrational Modes. *The Journal of Chemical Physics* **1996**, *105*, 4412–4426.

(4) Beck, M. H.; Jackle, A.; Worth, G. A.; Meyer, H.-D. The Multiconfiguration Time-Dependent Hartree (MCTDH) Method: A Highly Efficient Algorithm for Propagating Wavepackets. *Physics Reports* **2000**, *324*, 1–105.

(5) Worth, G. A.; Giri, K.; Richings, G.; Burghardt, I.; Beck, M. H.; Jäckle, A.; Meyer, H.-D. The QUANTICS Package, Version 2.2. 2020.

(6) Worth, G. A. Quantics: A General Purpose Package for Quantum Molecular Dynamics Simulations. *Computer Physics Communications* **2020**, *248*, 107040.
